# Use of rainbow trout skin treated with glutaraldehyde as a mesh for abdominal hernioplasty in rats

**DOI:** 10.1590/acb393024

**Published:** 2024-07-22

**Authors:** Carolina Seabra da Costa, Siria da Fonseca Jorge, Marcelo Abidu Figueiredo, Danielle Rangel Neves, Maurício Alves Chagas

**Affiliations:** 1 Universidade Federal Fluminense – Programa de Pós-graduação em Medicina Veterinária Clínica e Reprodução Animal – Niterói (RJ), Brazil.; 2 Centro Universitário Serra dos Órgãos – Curso de Graduação em Medicina Veterinária – Teresópolis (RJ), Brazil.

**Keywords:** Hernia, Abdominal, Surgical Mesh, Oncorhynchus mykiss, Glutaral, Herniorrhaphy

## Abstract

**Purpose:**

To test the use of rainbow trout skin as a surgical mesh in abdominal hernioplasties in rats.

**Methods:**

The experiment involved 20 Wistar rats receiving implants of trout skin processed for disinfection in 0.5% glutaraldehyde and preserved in 100% glycerin. The animals were divided into four groups, divided at 7, 15, 30, and 90 days postoperatively. Clinical and infrared thermography evaluations were performed, and after euthanasia, assessments of adhesion formations and sample collection for histological evaluation were conducted.

**Results:**

The implant was observed to be intact, ensuring the integrity of the abdominal wall, support for the viscera, and normal mobility for the rats for up to 90 days. Low rates of clinical alterations were observed, with an intense inflammatory reaction up to day 7, chronic inflammation and the onset of angiogenesis at day 15, and a low inflammatory reaction with collagenous infiltrate and fibrosis at day 30. At day 90, the implants showed a collagenous and fibrotic infiltrate with a minimal inflammatory infiltrate.

**Conclusions:**

The surgical mesh of trout skin performed well, making it a potential alternative for surgical procedures in muscle aponeurotic corrections in the abdominal wall.

## Introduction

Abdominal hernias are a prevalent disease that requires surgical intervention in medical practice^1^. The surgical management of abdominal hernias varies depending on the size of the hernial defect^2^. The selection of surgical techniques and the utilization of implanted mesh are directly correlated with procedural success. Attaining minimal postoperative reactions in hernioplasty procedures remains a contemporary surgical challenge^3^.

Despite the extensive literature on the subject, complications persistently arise with synthetic meshes, mainly due to their potential to incite a moderate foreign body reaction. This reaction can manifest in complications such as chronic pain, prolonged inflammatory responses, infections, seroma formation, and hernia recurrence, among others, whether explicitly mentioned or not^4^^,^ 5 ^,^ 6 ^,^ 7.

Biological surgical meshes, typically derived from animal tissues or corpuses, undergo various processing techniques, including decontamination and preservation, for subsequent utilization. The abundant presence of collagen in the extracellular matrix of diverse species imparts lower reactivity to biological implants, providing robustness and tensile strength. The interaction between these collagen fibers and the mammalian healing process establishes a cellular bond similar to natural tissue, resulting in inconspicuous scarring effects and potentially serving as a collagenous scaffold for adjacent tissues. Currently, biologically derived surgical meshes are applied to surgical sites with a history of infection or chronic infection and are under experimental investigation to develop readily available materials^6^^,^ 7 ^,^ 8 ^,^ 9 ^,^ 10.

Using fish and amphibian skin as the foundation for biological membranes is an expanding domain within regenerative medicine^10^^,^ 11 ^,^ 12 ^,^ 13. Fish skins facilitate cellular infiltration due to their absorbable nature and high collagen fiber content, expediting healing. Furthermore, rainbow trout skin contains ribosomal peptides with antimicrobial properties and a significant collagen content^14^^,^ 15 ^,^ 16 ^,^ 17.

Glutaraldehyde, a bactericidal disinfectant employed in the processing of biological membranes since the 1960s, is still studied for its efficacy as a pre-conservative treatment for xenografts, due to its capacity for cell stabilization^18^^,^ 19 ^,^ 20 ^,^ 21 ^,^ 22.

This study aims to assess the viability of rainbow trout skin, disinfected with glutaraldehyde and preserved in glycerin, as a biological surgical mesh in abdominal hernioplasty in rats.

## Methods

### Ethical standards

This experiment adhered to the ARRIVE guidelines^23^ and received approval from the Ethics Committee for the Use of Experimental Animals (CEUA/UNIFESO) at Serra dos Órgãos University Center, with registration number 528/21, in compliance with Law No. 11,794 of October 8, 2008 (Official Gazette of the Union, Brazil, 2008).

Twenty male Wistar rats (*Rattus norvegicus albinus*) with an average age of three months and weighing 300 ± 50 g were utilized. They were accommodated in the UNIFESO Science Facility in polypropylene boxes measuring 43 × 23 × 16 cm, furnished with autoclaved wood shavings, and containing toys. The rats were maintained under circadian cycle conditions, with a room temperature of 22 ± 2 °C, relative humidity between 45 and 60%, and air exchange at 10–15 air changes per hour. They were provided unrestricted access to Nuvilab^®^ complete commercial feed (manufacturer: Quimtia) and potable water ad libitum, replenished every 24 hours.

The rats were stratified into four groups based on the postoperative euthanasia period, at 7, 15, 30, and 90 days, with each group consisting of five animals. No control group was incorporated due of the Russell-Burch principles of “reduction, substitution and refinement” (3 R’s). This decision was informed by the extensive literature concerning biological implants of diverse origins, compositions, and processing methods, alongside the burgeoning exploration of fish skins as biological dressings, the comprehensive documentation of synthetic surgical mesh effects, and the potential for using a contralateral parameter in the same animal for thermographic analysis.

### Preparation of implants

The commercial agent, 2% glutaraldehyde (Glutaron^®^ 2% 1L, manufacturer: Rioquímica), was acquired and diluted in buffered distilled water with phosphate buffer saline (PBS) 1× 500 mL pH 7.4; manufacturer: Nova Biotecnologia) (pH 7.4) to the desired concentration of 0.5%^19^^,^ 20 ^,^ 21 ^,^ 22. Rainbow trout skins were obtained from a commercial breeding facility in Nova Friburgo - RJ, in partnership with the Serrano Regional Office of Rio de Janeiro State Fisheries Institute Foundation (FIPERJ), and processed in the Laboratory of Animal Origin Products (POA) at Centro Universitário Serra dos Órgãos (UNIFESO).

The fish were desensitized on ice until slaughter. After filleting, the skins were removed by friction with a knife and cleaned. Following, they were immersed in a 0.5% glutaraldehyde solution for 18 days^19^^,^ 20 ^,^ 21, rinsed with sterile 0.9% NaCl, and immersed in 92.8% alcohol for 10 minutes^24^^,^ 25. Finally, they were dried with sterile gauze and immersed in 100% glycerin for a minimum of 30 days^10^^,^ 26. Before use, the skins were rehydrated and submerged in sterile 0.9% NaCl for 20 minutes^10^^,^ 27.

### Anesthetic procedure

The anesthetic procedure was meticulously executed to mitigate animal stress^23^^,^ 28. Individual chemical restraint was achieved within an anesthetic chamber using isoflurane and 100% oxygen28. Subsequently, intraperitoneal administration of ketamine (Cetamin^®^ 10%; Syntec) (75 mg/kg) and xylazine (Xilazin^®^ 2%; Syntec) (10 mg/kg) was conducted^28^^,^ 29. Anesthetic maintenance was sustained via a face mask using the same gases28,29. Analgesia was administered subcutaneously with Tramadol Hydrochloride (Tramadon^®^ 50 mg/mL; Cristalía) (12.5 mg/kg) immediately postoperatively and continued for the subsequent five days^28^^,^ 29. Pain assessment was conducted using the Grimace scale, with rescue analgesia administered if necessary. For thermographic captures, chemical restraint was utilized, using an anesthetic chamber followed by a face mask administering isoflurane and O_2_ 27 ^,^ 28 ^,^ 30, with a return to consciousness typically within 1–2 minutes.

### Surgical procedure

**Figure 1:**
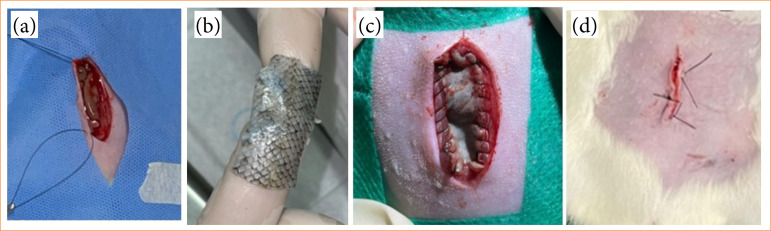
**(a)** Abdominal laparotomy through a xiphopubic incision in the abdominal wall; **(b)** Hydrated rainbow trout skin implant; **(c)** Implant fixed to the abdominal wall by continuous suture **(d)** Skin suture in *Wolf* pattern.

The surgical procedure entailed a midline xiphopubic laparotomy^10^^,^ 27. Following a skin incision, two sutures were positioned on the right side, followed by subcutaneous dissection and the creation of a defect in the right abdominal wall measuring 1.5 cm transversely and 3 cm longitudinally (Fig. 1a)^10^^,^ 26 ^,^ 27. Skin fixation was performed with the epidermis oriented towards the viscera, using a continuous simple interrupted suture at each edge of the wound (Fig. 1b and c), using a 4.0 nylon thread^10^^,^ 26 ^,^ 27. Skin closure was accomplished using an interrupted “Wolf” pattern with the same thread^25^^,^ 26.

### Postoperative care

Following surgery, the rats were transferred back to their respective cages. No anti-inflammatory agents were administered. Enrofloxacin (Zelotril^®^ 10%; Agener) was provided in the drinking water for 8 days^10^^,^ 27.

### Clinical analysis

The animals were weighed on the day of the surgical procedure and subsequently on days 3, 7, 15, 30, 60, and 90 postoperatively. Detailed observations comprise assessments of behavior, mobility, edema, seromas, hematomas, abscesses, fistulas, necrosis, and suture dehiscence. These parameters were graded on a scale from absent (-) to very severe (++++), following established protocols^10^^,^ 26 ^,^ 27.

### Thermographic analysis

Thermal images of the rats’ abdomen were captured using a Flir^®^ T420 thermographic camera with a resolution of 320 × 240 and sensitivity of 0.045 °C on the day of surgery and on days 3, 7, 15, 30, 60, and 90 postoperatively^10^^,^ 26 ^,^ 27. The images were processed using Flir Tools^®^ software, and average cutaneous temperatures on the right (implant) and left (control) abdominal sides were measured through ten parallel measurement points forming an imaginary craniocaudal line^26^^,^ 27. The average temperature on each side of the abdomen was then calculated for each animal and group.

### Macroscopic analysis

Following Conselho Nacional de Controle de Experimentação Animal (CONCEA) (2018) Resolution No. 37 and Law No. 11.794 of October 8, 2008, animal euthanasia was conducted employing chemical restraint within an anesthetic chamber using isoflurane and oxygen until unconsciousness was achieved. Subsequently, an intraperitoneal overdose of ketamine (180 mg/kg) and xylazine (30 mg/kg) was administered. Once fully anesthetized, with diminished vital parameters, potassium chloride was administered intracardially^30^^,^ 31.

Necropsy procedures involved making a U-shaped incision to facilitate the examination of the entire abdominal musculature, abdominal viscera, and any formed adhesions. These adhesions were classified on a scale as follows: Grade 0, indicating absence; Grade 1, representing mild adhesions (+), characterized by a count of adhesions less than or equal to 3, fibrinous in nature and easily disrupted by manipulation; Grade 2, indicating moderate adhesions (++), denoted by a count of adhesions greater than 3, firm and/or resistant to manipulation, predominantly found between intestinal loops without involvement of the abdominal wall; Grade 3, signifying severe adhesions (+++), characterized by firm adhesions resistant to manipulation, involving both the abdominal wall and organ or structure; Grade 4, indicating very severe adhesions (++++), marked by firm adhesions resistant to manipulation, observed between the intestinal loops and the abdominal wall, with the occurrence of enteric fistulas^10^^,^ 26 ^,^ 27.

### Histopathological analysis

Histological processing was conducted at the Cellular Biomorphology Laboratory of the Univesidade Federal Fluminense. Tissue fragments obtained from the muscle-implant interface of the rats and the entire implant were submerged in 10% buffered formalin. After standard paraffin embedding, histological sections of 5 μm thickness were prepared using a Leica RM125RTS manual microtome. These sections underwent staining procedures, including Hematoxylin-eosin (HE) and Masson’s trichrome. Photographic documentation was accomplished using a Leica DMLS30 microscope equipped with a Leica DFC425 digital camera, featuring ISO 200 sensitivity and a resolution of 1360 × 1024 at magnifications of 10, 20, and 40×.

The analyses were both descriptive and semi-quantitative, focusing on examining the interaction between rat tissue and trout skin implant, inflammatory infiltrate, and its spatial distribution. Additionally, secondary histological changes such as edema, hemorrhage, congestion, and angiogenesis were evaluated. ImageJ software (National Institutes of Health, Bethesda, MD, USA) was utilized to quantify the intensity of inflammation. Color segmentation was employed to identify pixels marked with the chromogen, determining their quantitative proportion over the total area analyzed. Mild inflammatory processes were defined as cases where up to 6% of the histological fragment comprised inflammatory cells, moderate when up to 15%, and intense when exceeding 15%.

### Statistical analysis

The data were analyzed using IBM SPSS statistical software. Descriptive statistics, including mean, difference, and percentage, were employed. The normality of weight and temperature data was assessed. Mean weight data were compared between groups on the day of the surgical procedure and the day of euthanasia using the Student’s t-test for paired samples. Similarly, mean temperature data were compared between groups during each postoperative period using the same test. Moreover, comparisons were made across analysis periods using the one-way ANOVA test.

## Results

### Clinical analysis

**Figure 2:**
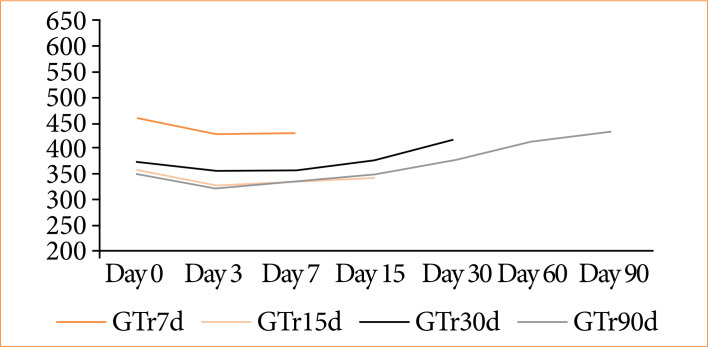
Average weight graph of animals per group across postoperative days.

**Figure 3:**
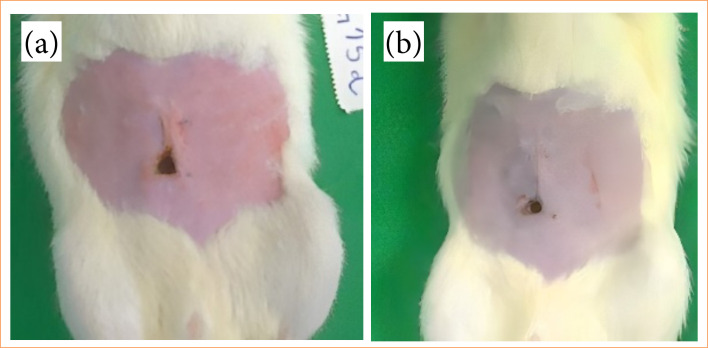
**a)** Suture dehiscence on day 7. **(b)** Evolution on day 15.

**Figure 4:**
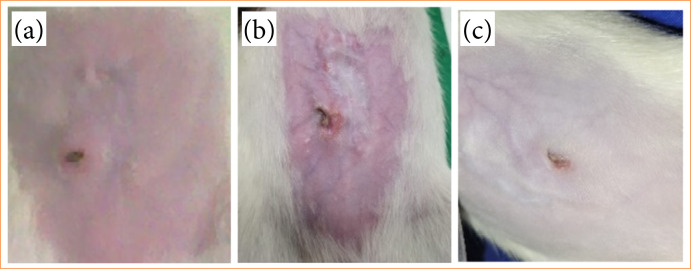
**(a)** Reaction to the internal suture thread on day 30. **(b)** Evolution on day 60. **(c)** Evolution on day 90.

The weight analysis of the animals revealed notable weight loss on the third postoperative day, followed by gradual recovery to baseline weight by day 30, with continuous weight gain thereafter (Fig. 2). A comparison of animal weights between the day of surgery and the day of euthanasia indicated an average weight loss of 28 g, or 6.12%, in the 7-day subgroup and 13.4 g, or 3.77%, in the 15-day subgroup. In comparison, weight gain was observed in the 30-day subgroup (44.4 g, or 11.96%) and the 90-day subgroup (81.6 g, or 23.28%). However, no statistically noticeable differences were observed in any of the analyses, individually, between the groups (p > 0.05). Clinical examination identified alterations in 2 out of 20 rats (10%). One rat exhibited suture dehiscence (5%; R2; 15 days) on day 7 (Fig. 3a and b). The other rodent presented a reaction to the internal suture thread (5%; R2; 90 days) on day 30 (Fig. 4), both displaying positive healing progression by second intention at the final evaluation (Fig. 4b and c). The remaining animals showed no signs of clinical alterations.

### Thermographic analysis

**Figure 5:**
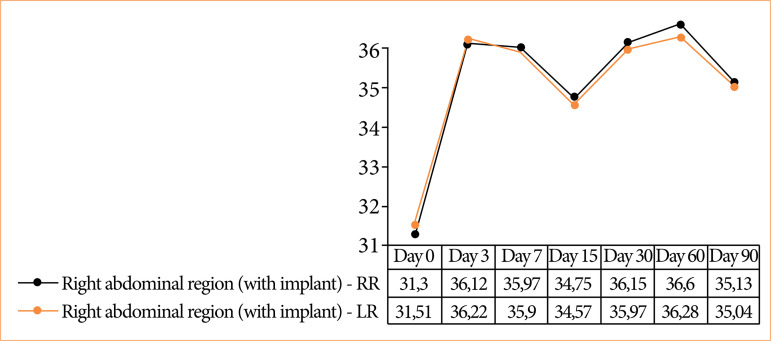
Demonstration of average temperatures on the right and left sides of the abdomen for all groups during postoperative periods.

**Figure 6:**
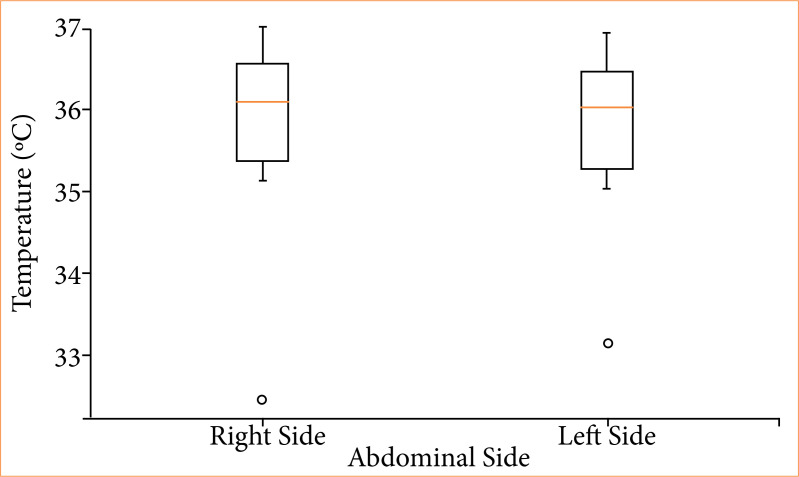
Temperatures on the right and left sides across the postoperative periods (p = 0.841).

Thermographic analysis demonstrated very low temperatures on day 0, with the left side having a higher temperature (0.28 °C). Subsequently, the left abdominal region showed a higher temperature on day 3 (0.09 °C). The right abdominal region showed a higher temperature in the following analysis periods: days 7 (0.032 °C), 15 (0.05 °C), 30 (0.14 °C), 60 (0.3 °C), and 90 (0.08 °C) postoperatively, as illustrated in the graphs (Fig. 5). No significance was observed in the statistical data processing between abdominal media in the groups: days 0 (p = 0.368), 3 (p = 0.63), 7 (p = 0.715), 15 (p = 0.264), 30 (p = 0.345), 60 (p = 0.32), and 90 (p = 0.464). Similarly, no statistical significance was observed in the analysis across the periods (p = 0.841) (Fig. 6).

### Macroscopic analysis

**Figure 7:**
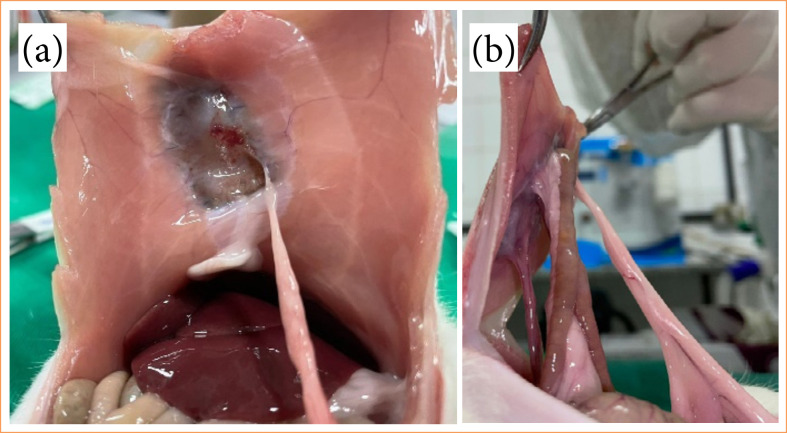
**(a)** Adhesions in LTL in suture. **(b)** Adhesions in RTL, omentum, and mesentery.

As shown in Table 1, adhesion formations were observed in all animals, with 85% categorized as Grade 1 or mild and the remaining 15% as severe. Thirty-one adhesion formations were recorded, as delineated in Table 1. Adhesions were notably prevalent, observed in 36% of the omentum, 39% in the right testicular ligaments (Fig. 7a), 6% in the left testicular ligaments, 6% in the intestine, and 3% in the mesentery (Fig. 7b). Remarkably, no fistulas or injuries to the implant and/or viscera were observed, even following manipulation and disruption.

**Table 1:** Adhesion formations in all animals.

Group (days)	Mouse	Number of adhesion	Adhesion structure	Adhesion location	Degree
7	1	1	Omentum	Suture	G1 +*
	2	3	Omentum, RTL*, Intestine	Implant	G3+++**
	3	2	Omentum RTL		G1+
	4	2	Mesentery, RTL		
	5	1	Omentum		
15	1	1	LTL**	Implant/Suture	G1+
	2	2	LTL RTL	Suture	
	3	3	LTL, RTL, Omentum		
	4	1	LTL		
	5	2	LTL, Omentum		
15	5	1	RTL	Implant/Suture	G1+
	2	1	LTL		
	4	1	RTL	Suture	
	3	1	Omentum		
	1	1			
	2	1	Intestine	Suture	G3+++
	3	2	RTL Omentum	Implant	G1+
	4	1	RTL	Suture	G1+
	1	2	RTL Omentum		
	5	2	RTL and Omentum		

* RTL: right testicular ligament.

** LTL: left testicular ligament.

### Microscopic analysis

**Figure 8:**
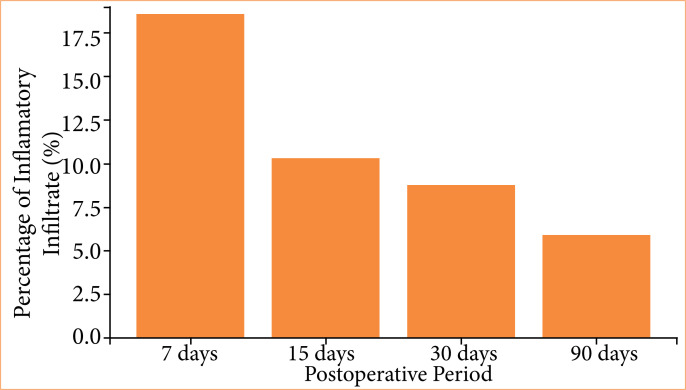
Intensity of inflammatory cellular infiltrate in groups, over postoperative days.

**Figure 9:**
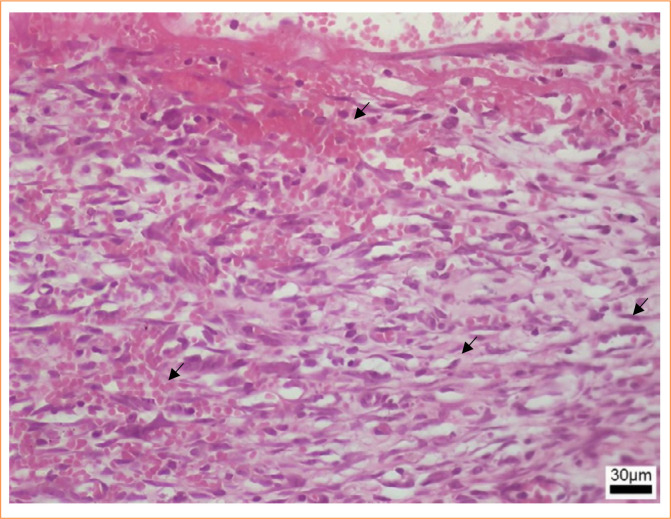
Photomicroscopy (40x): Intense inflammatory infiltrate with areas of hemorrhage (↙) and edema (↙).

**Figure 10:**
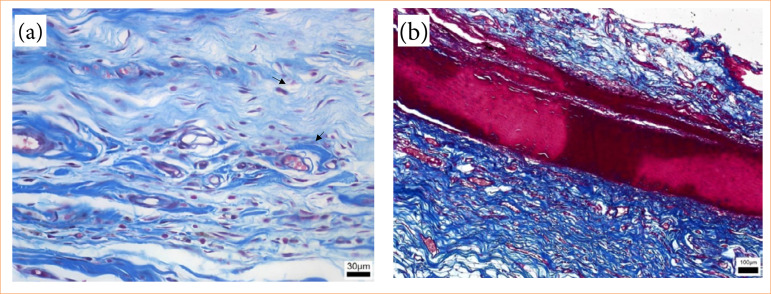
Photomicroscopy (40x): **(a)** Inflammatory infiltrate, areas of fibrosis (↙), and edema (↙); **(b)** 10x magnification: Implant in the early stages of delamination.

**Figure 11:**
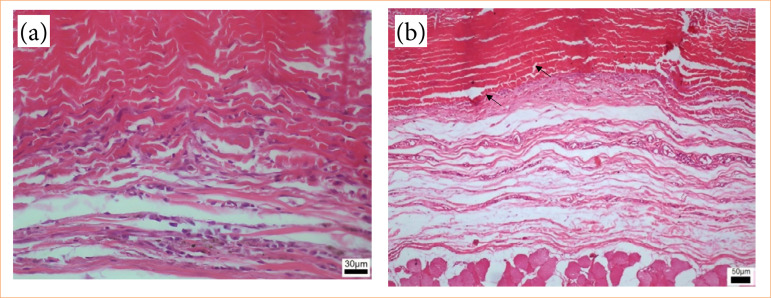
Photomicroscopy (40x): **(a)** Fibrinous tissue with mile cellular infiltrate and reduce of edema; 10x magnification: **(b)** Implant integrity with cellular and fibrinous tissue infiltrate, with the beginning of delamination (↘).

**Figure 12:**
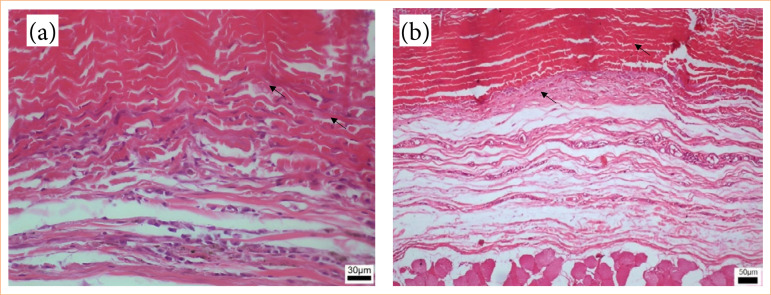
Photomicroscopy 40x: **(a)** Rainbow trout skin implant with low inflammatory infiltrate and clear delamination, with implant fibers interspersed with adjacent tissue (↖), as well as collagen fibers and fibrous tissue interspersed with the implant. 20x magnification: **(b)** Delaminating implant (↖) and low inflammatory infiltrate.

The inflammatory infiltrate exhibited greater intensity in the group euthanized on postoperative day 7, gradually diminishing in subsequent groups, presenting as moderate on postoperative days 15 and 30 and mild at 90 days (Fig. 8). The group euthanized at 7 days postoperatively displayed an intense inflammatory infiltrate (18.55%), evident through purple staining, alongside areas of hemorrhage and edema (Fig. 9). By day 15, animals demonstrated reduced inflammatory infiltrate (10.33%), with concurrent alleviation of hemorrhage and edema. Some focal points of fibrosis, dilated vessels, and capillaries were observed (Fig. 10a), along with the onset of the delamination process, characterized by cellular infiltrate at the lateral edges of the implant (Fig. 10b). At day 30, a further reduction in the inflammatory infiltrate (8.76%) was observed, along with focal areas of fibrosis (Fig. 11a). The implant exhibited some regions of infiltrated fibrinous tissue (Fig. 11b). At 90 days, a minimal inflammatory infiltrate (5.88%) was noted, along with the presence of collagen fibers and focal points of fibrosis (Fig. 12a). The implant remained intact, progressing through delamination (Fig. 12b).

## Discussion

The use of biological meshes for hernia repairs has been scrutinized in retrospective studies, with hernia recurrence emerging as the primary complication^7^^,^ 8 ^,^ 9, an outcome not observed in this experiment. Here, a comprehensive evaluation of the implant through thermography, necropsy, and histology revealed no abdominal bulging for up to 90 days. Concurrently, the animals exhibited normal behavior and body mobility, analogous to observations in studies employing both synthetic and biological meshes for midline musculofascial procedures. These findings underscore the robustness of the muscular wall stability and the physical efficacy of the implant^10^^,^ 18 ^,^ 20 ^,^ 26 ^,^ 27.

The weight dynamics observed in this study were consistent with those reported in investigations assessing implants derived from bullfrog and Nile tilapia skin26 and bubble wrap^27^ utilized as meshes in abdominal rat hernioplasties. The observed weight loss on the third postoperative day is primarily correlated to the anesthetic procedure, in which the administered drugs induce a state of thermal dysregulation, leading to intraoperative and postoperative hypothermia^28^^,^ 30. This phenomenon precipitates immediate metabolic alterations in small rodents^28^^,^ 29 ^,^ 30. Additionally, the surgical technique involving abdominal cavity manipulation and immediate postoperative trauma is also associated with body temperature loss^28^^,^ 29 ^,^ 30 ^,^ 31. The animals’ subsequent weight recovery, which surpasses baseline levels, underscores their restoration of endocrine functions and adaptation to the implant, facilitating movement for food intake and overall bodily development.

Experiments involving bovine pericardium implants in lateral abdominal muscle revealed cytotoxic effects of glutaraldehyde in concentrations of 1 and 1.5%, as evidenced by local reactions observed in necropsy for days 7, 15, and 30^20^. Although the concentration of glutaraldehyde in this experiment was lower, it is reasonable to assume that suture dehiscence may be correlated with its toxicity. Mild cytotoxicity has been reported in similar implants with lower concentrations, such as 0.65%^19^^,^ 20. Besides, the anchored skin suture pattern (Wolf) used in this experiment may have contributed to the observed reaction, as studies in rodents recommend anchored patterns for skin sutures to prevent individuals from reaching the knot by gnawing at the thread end^26^^,^ 29.

The reaction to the internal suture thread may also be correlated with the foreign body reaction induced by the biomaterial, particularly given its location and the high cellularity of the tissue adjacent to the implant^20^^,^ 29. The friction of the thread knot against the muscle in contact with the subcutaneous and hypodermis may have resulted in an exacerbated, albeit non-infectious, reaction, demonstrating satisfactory progression without intervention. Notably, abscesses and cutaneous and surgical site infections have been observed and reported in retrospective studies involving biological mesh in hernioplasties, often associated with prolonged hospital stays, extended antimicrobial use, and the necessity for prolonged recovery^4^^,^ 5 ^,^ 6 ^,^ 7 ^,^ 19 ^,^ 20.

The relatively low average temperatures of the animals on the day of the surgical procedure can be attributed to intraoperative and postoperative hypothermia, stemming from the anesthetic technique and surgical procedure^28^^,^ 29 ^,^ 30 ^,^ 31. Conversely, the higher temperature of the left abdominal region observed on the day of the surgical procedure and the third postoperative day may be correlated with surgical trauma in the adjacent region, characterized by intense inflammatory infiltrate and the absence of vascularization or cellular infiltrate in the implant. Notably, acute inflammatory cellular infiltrate is observed in the immediate postoperative surgical site, evident from the first-day post-surgery, characterized by infiltrated neutrophils^6^.

Infrared thermography is a valuable tool for capturing the radiation emitted by cellular movement in cutaneous tissue, immediate postoperative surgical trauma, and the body’s response to foreign bodies within biomaterials, which directly correlates with histological observations^10^^,^ 26. The documented increase in cellular activity, as indicated by higher temperatures on the side with the rainbow trout skin implant from the seventh day onwards, is attributed to cellular infiltrate within the implant and the initiation of angiogenesis^10^^,^ 25 ^,^ 26. Similarly, this trend aligns with observations in later periods, where the implant begins the incorporation process, as evidenced by histological findings. Late inflammatory reactions are well documented in studies using glutaraldehyde biological meshes alongside the concurrent processes of angiogenesis and implant incorporation^10^^,^ 19 ^,^ 20 ^,^ 21 ^,^ 26.

Furthermore, it was noted that even after 90 days post-surgery, the original collagen framework of the trout skin remained intact, indicating a slow absorption process. This characteristic proved to be advantageous for the material, facilitating the progression of the inflammatory process and subsequent healing while concurrently maintaining the stability of the wall due to the presence of the collagenous framework and cellular infiltrate. This phenomenon is corroborated by studies conducted within a 30-day period, which demonstrated regenerated bundles of muscle fibers surrounding the implant^20^^,^ 21 ^,^ 22. Additionally, preserving this collagenous framework during the incorporation process mitigates the risk of hernia recurrence, a phenomenon often associated with early absorption observed in studies involving biological meshes^7^^,^ 8.

Peritoneal adhesions are common in procedures involving the abdominal wall, irrespective of whether biological or synthetic meshes are utilized^5^^,^ 6 ^,^ 7 ^,^ 19 ^,^ 20. As evidenced in this experiment, adhesion formations were identified in all animals. These adhesions were observed in various anatomical locations, including the omentum, testicular ligaments, mesentery, and intestine. In the omentum, adhesions are a common occurrence with biological implants, and while they may not pose harm, they serve a functional role in combating infections and foreign bodies^26^^,^ 30. The observation of 100% adhesion occurrence in the omentum aligns with findings from previous studies involving both biological and synthetic implants^26^.

Adhesions in the testicular ligaments are attributed to the thermoregulation mechanism of rodents, as demonstrated in surgical studies involving abdominal wall procedures^10^^,^ 26. The proximity to the implant and the presence of mesothelial cells akin to vascular endothelial cells contribute to adhesion formation in the mesentery and intestine^7^. While adhesions in the intestine may pose complications if fistulated, no such rupture was observed in this study, consistent with observations from previous biological mesh studies that reported adhesions while preserving organ integrity^10^.

## Conclusion

The rainbow trout skin proves to be useful as a surgical mesh in abdominal hernioplasties. It effectively maintains the positioning of the muscular wall, supporting intracavitary viscera, and allowing normal movement in rats despite eliciting moderate reactions. However, considering the findings from studies using lower concentrations of glutaraldehyde, rainbow trout skin could be a suitable alternative for surgical mesh applications.

## Data Availability

All data sets were generated or analyzed in the current study.
